# From Footprints to Functions: A Comprehensive Global and Semantic Building Footprint Dataset

**DOI:** 10.1038/s41597-025-06132-z

**Published:** 2025-10-27

**Authors:** Laurens J. N. Oostwegel, Danijel Schorlemmer, Philippe Guéguen

**Affiliations:** 1 Section Dynamic Hazard and Earthquake Risk, GFZ Helmholz Centre for Geosciences, Potsdam, Germany; 2 ISTerre, Université Grenoble Alpes, Université Savoie Mont-Blanc, CNRS, IRD, Université Gustave Eiffel, CS40700 38058 Grenoble cedex 9, France; 3 Swiss Seismological Service, ETH Zurich, 8092 Zurich, Switzerland

**Keywords:** Geography, Natural hazards, Climate-change impacts, Civil engineering

## Abstract

Buildings play a critical role in understanding settlement patterns and are essential for crisis management, urban planning, energy efficiency, and multi-hazard risk assessment. To address the need for accessible global building data, we introduce a dataset containing 2.7 billion building footprints classified using the building taxonomy of the Global Earthquake Model. By conflating the AI-derived *Google Open Buildings* and the *Microsoft Global ML Building Footprints* datasets, and the crowd-sourced *OpenStreetMap*, we created the most detailed and extensive building dataset to date. This conflation helps balancing out the completeness bias in *OpenStreetMap* in which mapping is most complete in countries with high human developing index. We validated occupancy types and building height estimation using Kullback-Leibler divergence across specific cities, and through cadaster data from Slovenia and Greece, revealing that, while some misclassifications occur due to definitional differences or data limitations, the dataset overall provides reliable and valuable building information at global scale. Examples to use the data are to identify vulnerabilities of buildings for natural hazards or to model population distributions.

## Background & Summary

Buildings are at the center of many analyses, such as disaster response, urban planning or sustainability research. Whether one is calculating the energy consumption of a district, looking at mobility patterns in a city or estimating the extent of damage after a disaster in a region, building information is the key component. It is unsurprising that there is a dedicated field of research on identifying and classifying buildings and there are many models available that display building information. Raster datasets can be used for building density^1,2^. Building stock models aggregate building information per administrative area^3–6^. Building footprint models contain geometries of each individual building^7–11^. Nevertheless, we still do not know the location of all buildings on earth, let alone their type and size.

Official building registries can be the best source of building information, but are scarce and not always up-to-date^12^. Most countries do not have open building registries, either because of a lack of political will or financial resources^13,14^. Retrieving building information can even be complicated in countries with a strong economy. For example, the responsibility of the German cadaster lies within the individual states, leading to a separate dataset for each state^8^. This can result in incompatibility between datasets, both in format as well as in licensing.

*OpenStreetMap* is a crowd-sourced geographical dataset where one can map any geographical asset. As of 1 June 2025^15^, it contained 648 million building footprints, which may be tagged with additional details, such as the occupancy type, height, construction year or information like businesses residing the building and their opening times. It is created by individuals and organizations, at home or during mapathons, by imports, and through commercial, educational, humanitarian and government efforts. The diversity of information sources makes the dataset a great asset, but it also exposes its own biases: (1) in practice the information in *OpenStreetMap* is densest in the Western world^16–18^, so one cannot rely on the dataset to be complete in an arbitrary area. (2) The unstructured nature of the dataset—where features can be mapped in various ways—can create inconsistencies. While there are conventions that should be followed to create some structure, these are not always adopted, due to divergent local conventions, time restrictions of the mapper or lack of experience.

Recent advances in artificial intelligence (AI) offer new possibilities for mapping buildings. For several years, AI technologies have enabled the automated derivation of building footprints from satellite imagery^9,19,20^. Although image recognition and even building footprint detection using Machine Learning (ML) methods have been in use for many years^21–23^, in the recent AI boom the scale of the published datasets increased to a global level. Datasets of such scale typically only contain building footprints, with no additional attributes. At times, an estimation of building height is provided. The vast scale of these datasets proves useful in giving an initial estimation of how many buildings there are to be found in a region. The information is of limited use, as no semantic information is known and the quality of AI-derived datasets generally is worse than of *OpenStreetMap*. An initiative from *Google*, Open Buildings (hereafter called the *Google* dataset), consists of 1.8 billion buildings, about three times the amount compared to the *OpenStreetMap* dataset. The *Google* dataset spans across South- and Central America, Africa, India, and South-East Asia^9^. The Global ML Building Footprints, an initiative from *Microsoft*, is a global-scale product (hereafter called the *Microsoft* dataset). It contains 1.4 billion buildings and is regularly updated, with its latest update on 28 February 2025^10^. The *Microsoft* dataset contains height information in parts of Europe and the USA. Both datasets are published with a license that is compatible with integrating their data into *OpenStreetMap*. Nevertheless, the *OpenStreetMap* community has made it clear that the automated incorporation of the AI datasets into *OpenStreetMap* itself is not desired for the foreseeable future: the geometric quality is not deemed to be sufficient, and there are too many false positives to import such datasets. These concerns come on top of the general issues inherent to imports into *OpenStreetMap*^18^. There are also other AI generated datasets available, such as the East Asian buildings dataset, that also has been published under an open license. However, since the data is derived from Google Earth, the validity of the license is unclear, therefore we ignored this dataset^20,24^.

The advent of global building footprint datasets derived using AI methods raises the question of whether such datasets can be used in addition to the maps created and curated by volunteers. Setting aside the inevitable inaccuracies the AI methods bring, the datasets can improve the level of information that is crucial for understanding the built environment (location, size and type of buildings). The location of buildings can be found *en masse* in the *Google* and *Microsoft* datasets, which both contain more than twice as many building footprints compared to *OpenStreetMap*. On top of that, the *Microsoft* dataset provides the height of the buildings in meters in some regions, consequently giving us the ability to make assumptions on the size of buildings. AI algorithms and datasets could be key to overcome the issues of the coverage of the *OpenStreetMap* dataset^25–27^. An example of a dataset following this approach is *Overture Maps*, which, instead of merging building data from *Google* and *Microsoft* into *OpenStreetMap*, created a separate conflated dataset. Even though the schema of *Overture Maps* is well documented^28^, there is no clear description of the dataset construction or data quality.

We created *OpenBuildingMap*, a building footprint dataset that minimizes the disadvantages of using either *OpenStreetMap* or AI-derived datasets. For data users, *OpenStreetMap* can be hard to use because of the lack of a clear structure and ontology. AI-derived datasets suffer because of their limited semantic information and data quality. *OpenBuildingMap* combines the richness of the *OpenStreetMap* dataset with the extended coverage of the AI datasets. It conflates multiple building footprint datasets, such that it is the most complete footprint dataset to date with preference of the highest quality building geometries of the input datasets. It includes the building geometry, the floorspace, height in meters, number of stories and occupancy type of the building where possible and provides these in a structured way, with a clear denomination for all semantic data and available openly in an easy-to-access data format.

*OpenBuildingMap* can be used in a wide range of applications. Urban planners can use the dataset for city modeling, or assessing urban growth patterns. Disaster management organizations may do risk assessments, evacuation planning, or damage estimation following natural disasters using the building information. The occupancy type and building size can be used to estimate at what time of the day the building is occupied and the number of occupants inside the building. The dataset can be used in sustainability analysis, by looking into housing heating systems or feasibility of solar panels on roofs, using the building footprint size and height information. Other applications can be found in fields such as economics, sociology, and computer vision.

## Methods

**Fig. 1:**
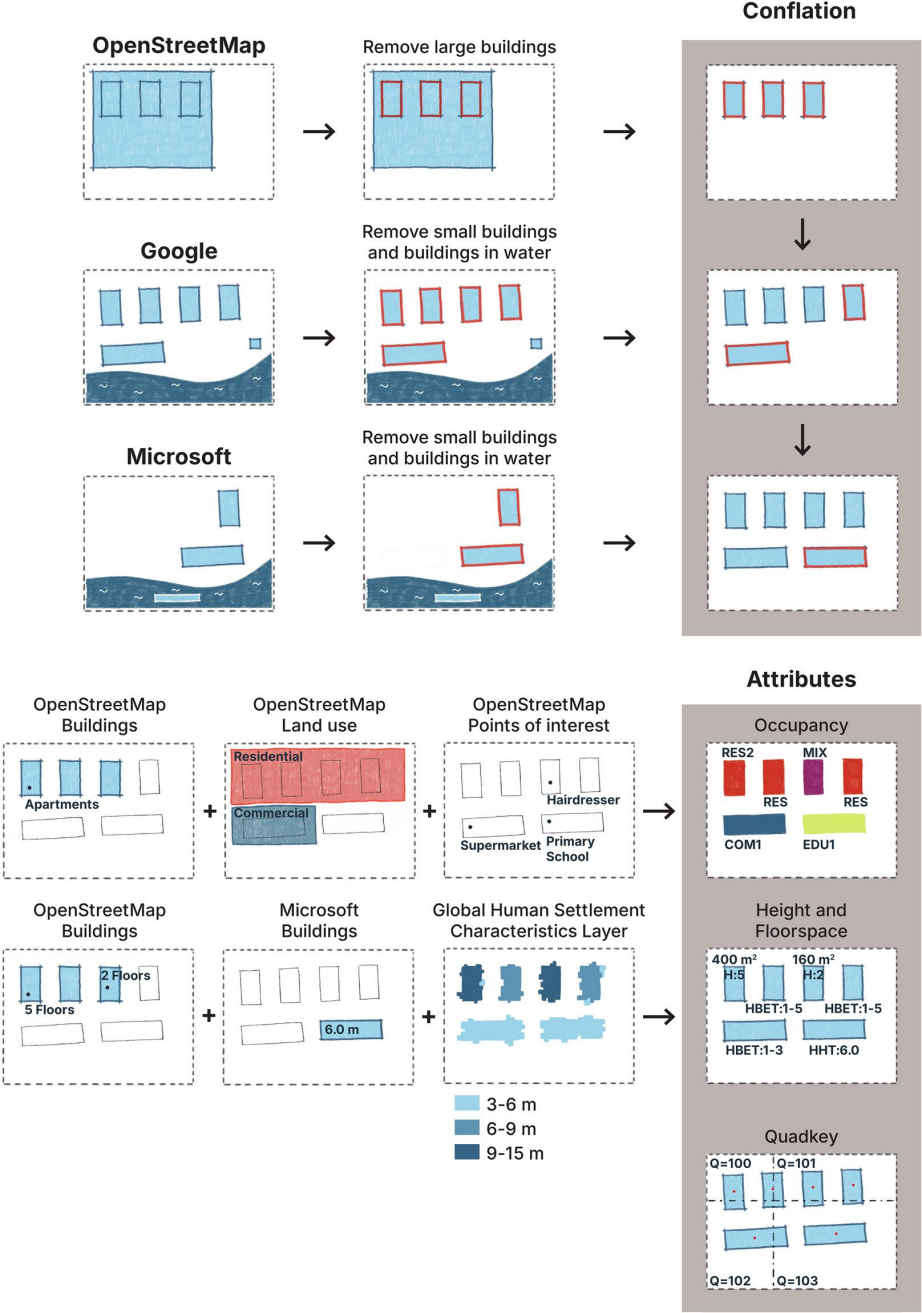
Overview of the methods. All buildings in the *OpenStreetMap* dataset with a footprint size bigger than 200.000 *m*^2^ are manually reviewed and deleted if they are incorrectly classified as a building. All buildings in the *Google* and *Microsoft* datasets that are in water or with a footprint size smaller than 6 *m*^2^ are deleted. The datasets are conflated based on precedence (in the order *OpenStreetMap*, *Google*, *Microsoft*). The building occupancy is added based on the building attributes, the *OpenStreetMap* points of interest and *OpenStreetMap* land use. The building height is added based on the building attributes and *GHSL* and the floorspace is computed, if the number of stories is known. The Quadkey is added based on the coordinates of the building centroid.

The development of the *OpenBuildingMap* dataset followed a three-fold method, that is elaborated on in this section. First, we conflated all building footprints from the three open building datasets: *OpenStreetMap*, *Google* and *Microsoft*. A precedence was followed based on the quality of the datasets, to avoid building duplicates. Second, four attributes were derived using the individual building attributes and information from other datasets, such as land-use information from *OpenStreetMap*. We estimated the number of stories or height information where possible, using either building attributes from the respective datasets or from the Global Human Settlement Characteristics Layer (hereafter *GHSL*)^2^. Using the number of stories and the building footprint, we estimated the usable floorspace. We also acquired information from *OpenStreetMap* in and around the building footprints, such as land use information and points of interest (e.g. restaurants, schools, or hospitals) to deduct the occupancy type. The occupancy and height attributes were classified according to the definition of the building taxonomy of the Global Earthquake Model^29^, a standard used in earthquake risk. A locational identifier (Quadkey) was computed using the building centroid. Third, the resulting dataset was validated, using reference datasets where possible and intrinsic methods for values where no reference is available. The overview of the method is visualized in Fig. 1.

A concept that will come back many times is the Quadtree system^30^. In this system, the globe, projected onto a square using the Pseudo-Mercator (also Web Mercator) projection, is recursively divided into four squares, such that the north-west square is assigned the number 0, the north-east square the number 1, the south-west the number 2 and the south-east the number 3. We define the zoom-level of the Quadtree grid as the number of times the grid has been sub-divided. A Quadkey is the unique identifier inside that grid, where each digit from left to right signifies the quadrant at that given zoom-level. The Quadkey 23 identifies the south-eastern quadrant at zoom-level 1 (the 3) inside the south-western quadrant at zoom-level 1 (the 2). In a zoom-level three grid that divides the globe into 4^3^ (64) tiles, the Quadkey 000 is the most north-western tile. The tile to the east of this one is the Quadkey 001 and the tile to the south is the Quadkey 002. It should be noted that the Pseudo-Mercator projection introduces a distortion towards the poles and that the size of Quadkeys around the equator are bigger than Quadkeys of the same zoom-level around the poles.

### Input datasets, data quality and data cleaning

The *OpenStreetMap* dataset is a collection of geographic information from volunteers using an open data scheme: anybody can add geographic features (relations, ways/polygons, nodes) and add tags to these features. Buildings are polygons with the tags building or building:part, or where the tag aeroway has the value terminal or hangar. If the building tag is of any of the values: no, none, bridge, pier, road, dam, ruins, ruin, construction, roof, collapsed, foundation, wall, they will be ignored. The buildings are imported into a *PostGIS* database, using the Osmium^31^ tool. The Osmium mapping file that has been used to define which *OpenStreetMap* features to import into the *PostGIS* database, as well as which attributes should be converted to columns, is found in the data repository.

We performed some basic quality filtering on the *OpenStreetMap* dataset. Numerous studies have shown that the base quality of *OpenStreetMap* is sufficient for disaster-risk related usage, with the main limitation being the completeness of the data^32,33^. The coordination of humanitarian mapping in *OpenStreetMap* usually happens within the HOT Tasking Manager, that has a built-in review process. Following the tasking manager, each building that is mapped should be seen by at least two pairs of eyes (a mapper and a reviewer). On top of that, mapping software like JOSM (one of the most used tools for mapping *OpenStreetMap* features) include quality assurance tools. Nevertheless, in a mapping effort of this size, errors exists, created on accident or sometimes deliberately. The most problematic error frequently found in the dataset is adding the tag building=yes, instead of landuse=residential to entire areas containing buildings, rather than to individual buildings. It is likely the case that novice mappers add residential building zoning, therefore they add the tag building=yes. It has some major implications for the *OpenBuildingMap* dataset, as these areas can potentially take up full city districts. For this reason, we have manually checked all features tagged as buildings above 200.000 *m*^2^, roughly the size of the biggest buildings in the world. In total, there were 554 buildings that exceed the area threshold, of which 375 were correctly identified buildings, 144 were land-use polygons and 35 were non-identifiable polygons. The land-use polygons were changed in *OpenStreetMap* to landuse=residential, while the non-identifiable polygons were removed. We also removed building-story tags from *OpenStreetMap* above 175 (the Burj Kalifah, currently the highest building in the world, holds the record of the highest number of floors in a building with 163). There were in total 70 buildings that exceeded this threshold.

The *Google* and *Microsoft* datasets are constructed using convolutional neural networks and high-resolution satellite imagery from *Google* satellite imagery and *Bing* satellite imagery^34^. The *Google* dataset covers large parts of Central and South America, Africa, India and South-East Asia. The *Microsoft* dataset covers most of the world, with the biggest blind spot being China. However the coverage is not as high within countries, compared to *Google*. For example, India contains 151 million buildings in the *Microsoft* dataset, as compared to 514 million in the *Google* dataset. Even though the buildings in the *Microsoft* dataset are scattered around the entire country, many buildings that exist in the *Google* dataset are not detected. Datasets constructed using AI are expected to have falsely identified buildings, that in reality are e.g. parking places, white rocks, open mining pits or boats. The buildings from the *Google* and *Microsoft* datasets are imported into a *PostGIS* database with our own *all-in* tool (see code availability). The *Microsoft* dataset has a height attribute in parts of Northern America, Europe and Australia, that defines the building height above ground level and is imported too.

**Fig. 2:**
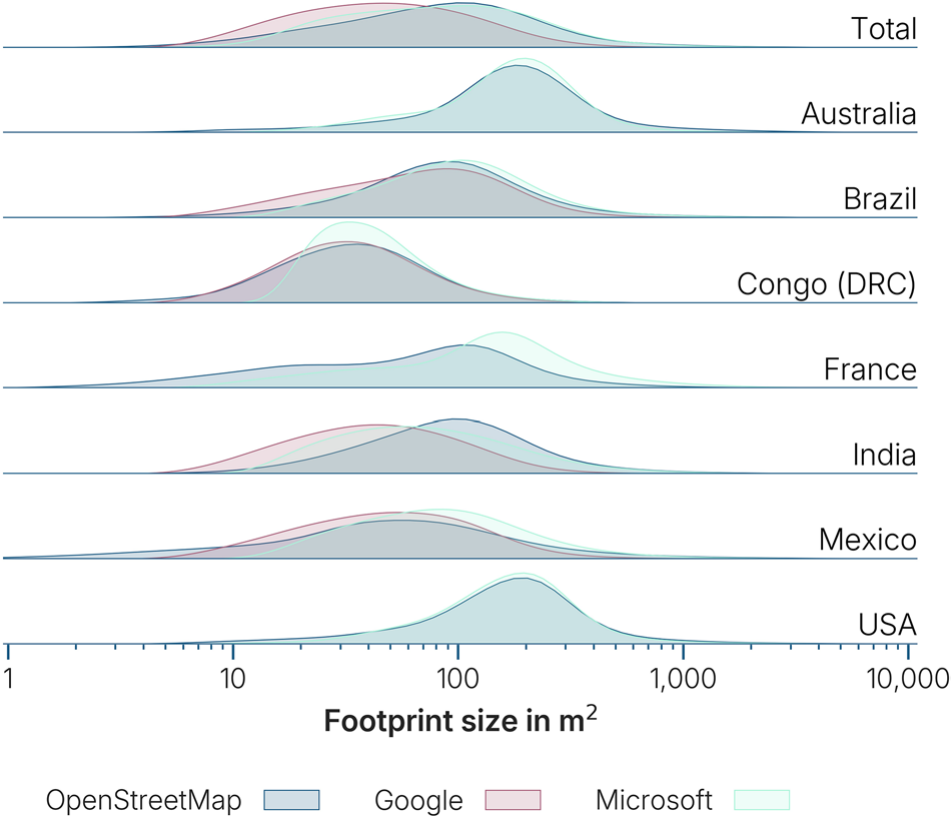
Distribution of building footprint sizes in *OpenStreetMap*, *Google* and *Microsoft*. The *Google* dataset is overall having smaller footprint sizes than *OpenStreetMap*, while the *Microsoft* dataset shows in some cases (France, Congo, Mexico, Australia) bigger footprint sizes than *OpenStreetMap*. The latter is more problematic, as many building footprints get joined into one single building footprint. The resulting feature could include the streets dividing the joined buildings, therefore overestimating the built area.

**Fig. 3:**
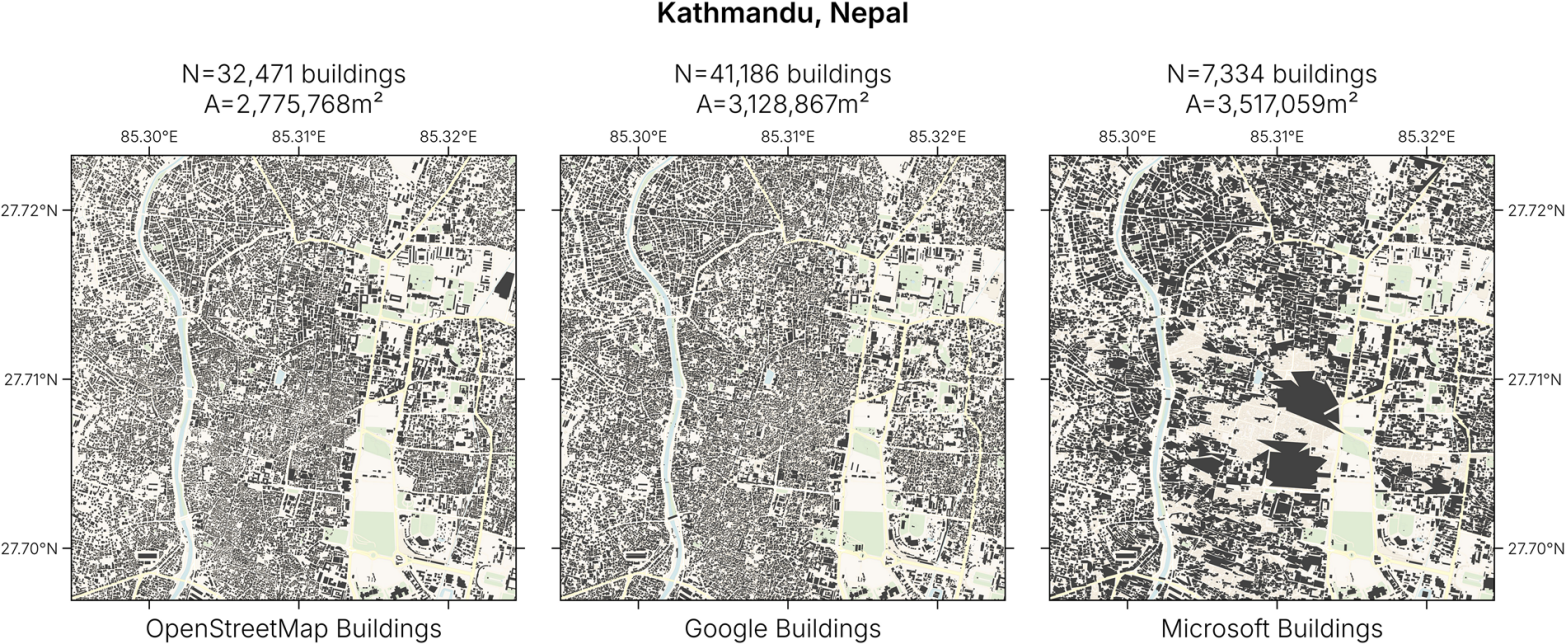
Comparison of footprints in the inner city of Kathmandu, Nepal. The footprint geometries and number of buildings (*N*) in the *OpenStreetMap* and *Google* datasets are relatively similar, while the *Microsoft* dataset shows large aggregated buildings. Even though there are four times fewer buildings, the total footprint area (*A*) is higher in the *Microsoft* building dataset than in the other two datasets. ©Basemap by CARTO and OpenStreetMap contributors.

Quality filtering was also needed for the *Google* and *Microsoft* datasets. False positive detections included geometries in water (ocean, river or lake), or very tiny geometries. Cargo boats were frequently wrongfully detected as a building as well as rocks along seashores. For example, along the coast of Spitzbergen, more than 340,000 ‘buildings’ are found in the water in the *Microsoft* dataset. Therefore, all buildings found in water bodies, as mapped in *OpenStreetMap*, were removed from the AI datasets. This also removes houses raised on stilts and boat houses, but as there are so many false positives, the total error is reduced. On top of that, many false positive buildings were of a particularly small size. We removed all buildings smaller than six square meters, roughly the size of the K67 kiosk^35^. We define this kiosk to be the smallest possible unit that can be identified as a building. Below this size, features are frequently misidentified. For example, most features that we found in the water near Spitzbergen were below 6 square meters. It has been reported that buildings in the *Microsoft* dataset are generally larger than the buildings in the *Google* dataset and encapsulate multiple buildings into one, while the *Google* dataset detects more buildings^34^. The *Google* dataset also has less missing buildings and false positives than *Microsoft*^25^. We can see this pattern on a global scale too. On average, the size of building footprints in the *Google* dataset are smaller than in the *Microsoft* dataset (Fig. 2). Even though there are fewer buildings, the *Microsoft* dataset totals a higher amount of built area than the *Google* dataset in the same area. A visual inspection of the *Microsoft* dataset shows a pattern of building blobs that do not represent reality, but rather aggregate many buildings into jagged geometries (see for example Fig. 3 from the city center of Kathmandu, Nepal). Such errors are less common in the *Google* dataset, where more buildings are detected in comparison to the *Microsoft* dataset, that have a smaller footprint.

### Conflation of building features

Many building footprints are duplicated in two or three of the datasets. These duplicates usually do not have a perfect overlap. This is expected, as not only different methods are used to construct the datasets; it is likely that for each of the datasets, different satellite imagery has been used. Next to that, what may be considered ten separate buildings in the one of the datasets, could be identified as a single building in another dataset. Lastly, all building footprint sources differ in their geographical coverage and completeness. Considering the aforementioned differences in the datasets, we opted for a simple conflation method, where non-overlapping buildings are added based on a priority of the source dataset.

The conflation of the building footprints followed the precedence: *OpenStreetMap*, *Google*, *Microsoft*. We expect the least errors in the building footprints with buildings from the *OpenStreetMap* dataset. We have seen that the *OpenStreetMap* dataset has its flaws, as being constructed by a large group of volunteers (e.g. misclassifications). On the other hand, all incoming information is overseen by the human eye, rather than an algorithm. The *OpenStreetMap* dataset is also most up-to-date, with minutely edits, while the other two datasets are solely based on satellite imagery that could be more than 10 years old (the *Microsoft* dataset is based on satellite imagery between 2014 and 2024^10^. Therefore, we first added all *OpenStreetMap* buildings to the building dataset. We have also seen before that the quality of *Google* is better than of *Microsoft*. Therefore, all *Google* building disjoint with the *OpenStreetMap* buildings were added next. Lastly buildings from the *Microsoft* dataset were only added, if they were disjoint from both other datasets.

### Addition of semantic information

The addition of semantic information in this study can be grouped into three categories: (1) building attributes, (2) contextual information from *OpenStreetMap*, and (3) contextual information from *GHSL*.

Building-specific attributes were sourced from both the *Microsoft* dataset and *OpenStreetMap*. The *Microsoft* dataset only provides building height information. Due to the open data structure of *OpenStreetMap*, the availability and specificity of attributes varied significantly: the building type, height, and the number of stories were present to varying degrees. In *OpenStreetMap*, buildings can be related to other features (relations). For example, a single building footprint is part of a school complex. In such cases, attributes were also derived from the tags of the related features.

*OpenStreetMap* data were also used to derive contextual attributes, particularly points of interest (POIs) and area features. POIs are node features with values that had any of the keys: aerialway, amenity, building, historic, landuse, leisure, office, or shop. The area features are polygonal features with curated values that had any of the keys: amenity, landuse, leisure, natural, place or tourism. These values were used to infer building occupancy types. For example, a node labeled amenity=school suggests an educational use, while a polygon tagged amenity=place_of_worship indicates that the buildings within it are related to religious activities. A full list of keys and values is provided in the data repository.

**Fig. 4:**
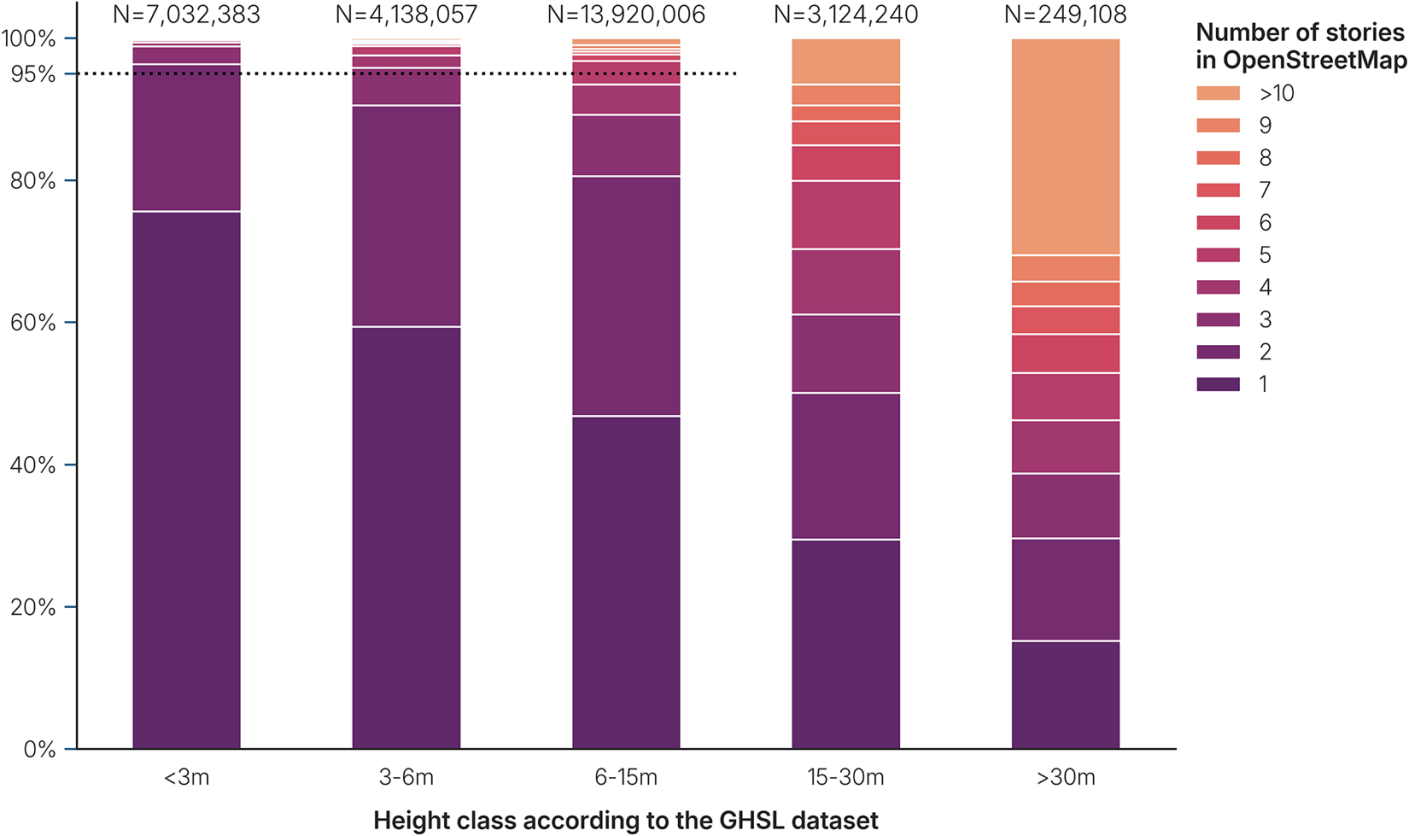
Composition of number of stories in *OpenStreetMap*, as compared to the height in *GHSL*. More than 95% (dotted line) of the buildings with a height below 3 meters, are between 1–2 stories high. Similarly, buildings classified between 3–6 meters are between 1–3 stories high. Buildings classified between 6–15 meters, are mostly between 1–5 stories. The distribution of number of stories of buildings classified between 15–30 meters or higher is too dispersed to give any estimate.

In cases where building height was unavailable in the building datasets, we used the *GHSL* dataset to estimate height. This is a global raster dataset with a resolution of 10 by 10 meters and pixels classified into a type (residential building, non-residential building, road, water, vegetation) and height, in case of a building type (<3 meters, 3–6 meters, 6–15 meters, 15–30 meters, >30 meters). Because the dataset provides only ranges of height rather than precise values, we used it to estimate ranges of building stories. We first analyzed 32 million buildings with a known number of stories in *OpenStreetMap* to establish correlations with *GHSL* building height classifications (see Fig. 4). Based on these correlations, we estimated that buildings with heights below 3 meters (from the *GHSL* dataset) were typically one to two stories tall. In reality, no two-story building would be less than three meters high, but in such cases *GHSL* underestimates the height of the building. The dataset provides only an indication of a height class, rather than a floating point value^2^ and is not always accurate. All-in-all, heights between 3 and 6 meters were associated with 1–3 stories. Heights between 6 and 15 meters were more dispersed and corresponded to 1–5 stories. However, for buildings with heights exceeding 15 meters in *GHSL*, the variability was too large to make useful and reliable estimates of the number of stories. This is in line with previous research, that finds that the inter-story height of buildings highly varies^36^. The higher the building in meters above ground, the more difficult it becomes to estimate the number of stories.

### Validation methods

It is not trivial to validate the results, as an external reference dataset is not available. The cadastral building footprints of countries with an open cadaster may already be imported in the *OpenStreetMap* dataset, as is the case for the Netherlands and France. In these cases it does not make sense to compare with a cadastral reference dataset, because the conflated dataset is identical. On top of that, cadastral information is only available in regions, where *OpenStreetMap* is generally of better quality too^17^. This can give a skewed view of the quality of the resulting dataset. We chose three methods to assess the validity of the dataset: (1) estimation of completeness of the building footprints using the *GHSL* dataset; (2) an intrinsic method to calculate the quality of the occupancy type estimation using entropy in urban areas; and (3) a comparison of cadaster buildings in multiple areas with the *OpenBuildingMap* dataset. We selected two locations (Greece and Slovenia), where there is an existing cadaster that includes height and/or occupancy information, but it is not imported into the *OpenStreetMap* dataset.

#### Completeness assessment

We assess the completeness of the conflated building footprints by comparison with the built area from *GHSL*. Research on an older version of the built-up area in the *GHSL* dataset shows that rural settlements are detected well, while it is not overestimating the built-up area as much as other settlement layers such as the *ESRI land cover*^37^. We have used an improved *GHSL*, that has been released in 2023. The comparison between the results of the conflation and *GHSL* is a crude measure because *GHSL* still overestimates the total square meter of built-up area in some regions and may miss built-up areas somewhere else^38^. However, unlike the *OpenBuildingMap* dataset, the *GHSL* dataset is homogeneous in coverage. Systematic under-representation of buildings in the conflated dataset compared to *GHSL*, can indicate a lack of building coverage in the area.

Despite *GHSL* not exhibiting the same degree of overestimating as other settlement layers, there is still a noticeable overestimation in built-up areas^37^. Hence there is no point to make a precise comparison between the area of the building footprint and the area represented by *GHSL*. Instead, we looked at the presence of either *GHSL* or *OpenBuildingMap* footprints on a zoom-level 18 Quadtree grid. The proportion of tiles with solely *GHSL* features present in comparison to the total number of tiles in a zoom-level 12 grid can provide an estimation of the (in)completeness within an area. To illustrate, we can assess the presence of geometries in a grid of 100 × 100 meters and assess the completeness on a 1 × 1 kilometer tile. If out of all 100 smaller tiles, 90 either contain buildings with the reference data, or only buildings, while 10 only contain the reference data, the completeness index is 90%. This does not necessarily indicate that there is a 90% coverage (since *GHSL* overestimates the built area), but it does enable to compare countries or areas with each other. If we know which countries in *OpenStreetMap* have a complete coverage of building footprints, we can expect other countries that have a complete coverage to have a similar completeness index, since the settlement layer has a similar coverage globally.

#### Quality of the occupancy type estimation

We estimate the occupancy type of a building by several means, such as the attributes of the building, POIs and land use information. This approach works well in areas with rich data. For example, if a building is located in a residential land use zone but is augmented with a POI indicating a church, it would be classified as a church. However, in regions with sparse data—where only land use is available—buildings may be misclassified. For instance, a church within a residential land use zone might be wrongly categorized as a residential building, if no POI identifies it as a church. Since no reference dataset exists to verify each building’s classification, we cannot simply check how many churches are misclassified.

In general, we can observe that areas with a higher quality of mapped data result in a more heterogeneous set of occupancy types. This means that the entropy in regions could be somewhat correlated to the quality of the data. One could use Shannon entropy^39^, which reaches its maximum when all building types are equally represented. Such scenario is unrealistic, as certain building types (e.g. residential buildings) will naturally dominate other, less common building types (e.g. churches). To address this, we propose comparing the distribution of occupancy types with an expected distribution, that can be constructed using cities that are known to have rich information in OSM. We employ the Kullback-Leibler divergence to quantify how much an observed distribution deviates from the expected distribution. If the similarity of two distributions is low, the divergence becomes higher. A high divergence therefore could indicate that it is unlikely that an observed distribution reflects the ground truth well. The Kullback-Leibler divergence is defined as follows in equation (1):

1 $${D}_{{\rm{KL}}}(P\parallel Q)=\sum _{x\in {\mathscr{X}}}P(x)\,\log \left(\frac{\,P(x)}{Q(x)}\right),$$

where P is the distribution of occupancy types in a city, Q is a reference occupancy distribution, x is the relative occurrence of one occupancy type in an area over the total set of occupancy types X^40^.

A measure as such can only be used if two key assumptions hold: (1) the observed region should have at least some degree of heterogeneity in building types; and (2) the real situation in the areas that are compared should have a similar occupancy type distribution as the expected one. Otherwise, meaningful comparisons are not possible. Metropolitan cities, particularly their city centers, are diverse in their building occupancy types. This differs in suburbs, villages or large industrial areas, where the same type of building is found over and over again, making them unsuitable for this method as they fail assumption (1). Additionally, metropolitan areas can vary significantly in size, with their outskirts resembling suburban regions. To ensure assumption (2) is met, we limit our analysis to a radius of 5 kilometer from the city center within metropolitan areas.

**Fig. 5:**
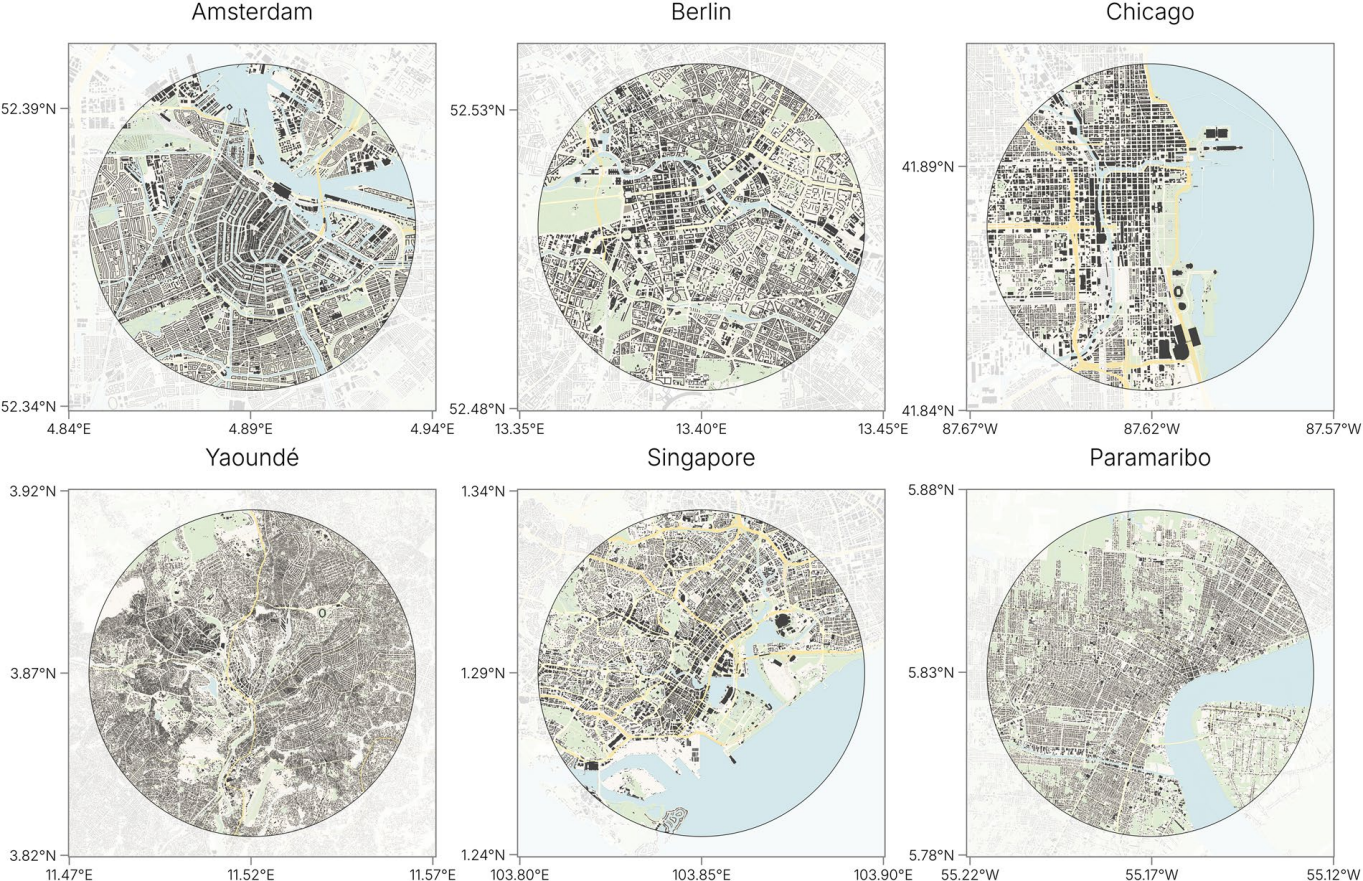
Buildings within a radius of five kilometers from the city center of the six cities that are used to calculate the expected occupancy distribution. ©Basemap by CARTO and OpenStreetMap contributors.

The expected distribution of occupancy types is constructed with the cities: Amsterdam (Netherlands), Paramaribo (Suriname), Vienna (Austria), Chicago (United States), Yaoundé (Cameroon) and Singapore (See Fig. 5). The number of unknown building types is excluded in the Kullback-Leibler analysis, but calculated as a separate value of quality. The center point of the city is taken from the administrative center nodes of the cities as defined by *OpenStreetMap*.

#### Case study: Greece and Slovenia

We have used two case studies to validate the individual building height and occupancy types in detail. In the case studies, we have compared cadastral information with the resulting *OpenBuildingMap* dataset. The locations are selected based on the following requirements: (1) The OSM-sourced building data should not have been inserted by import from the cadaster, as the comparison should be achieved with independent data. Therefore it is not possible to use countries like the Netherlands or France, where the entire building dataset is imported from the cadaster. (2) The conflated building data should not contain only one source, but multiple: we want to validate the dataset where there is not only the *OpenStreetMap* dataset, but also where AI-derived buildings are found. Consequently, Germany and many islands are not fit for validation for this reason. (3) The cadastral information not only needs to be available in the region, but should hold rich semantic information as well, so that height and/or occupancy type information can be validated. These three requirements limit our search greatly.

The Slovene cadaster is published with a CC BY 4.0 license and contains multiple height-related attributes. It contains one dataset with 1.2 million building centroids, of which 99% have a known number of stories and 94% have both the minimum height and maximum height in meter above sea level, that can be used to deduce the height of the building in meters. There has not been a big import into *OpenStreetMap*, therefore it is an ideal candidate to validate the height attribute of the *OpenBuildingMap* dataset.

The Greek cadaster contains 6.5 million parcels with parcel types. It does not have a complete coverage and about 36.5% of the occupancy types are unknown. The parcels include non-building types like road types and agricultural areas. There are 68 parcel types, of which 48 could be related to building occupancy types. Any other parcel type has been identified as ‘other occupancy’.

## Data Records

**Fig. 6:**
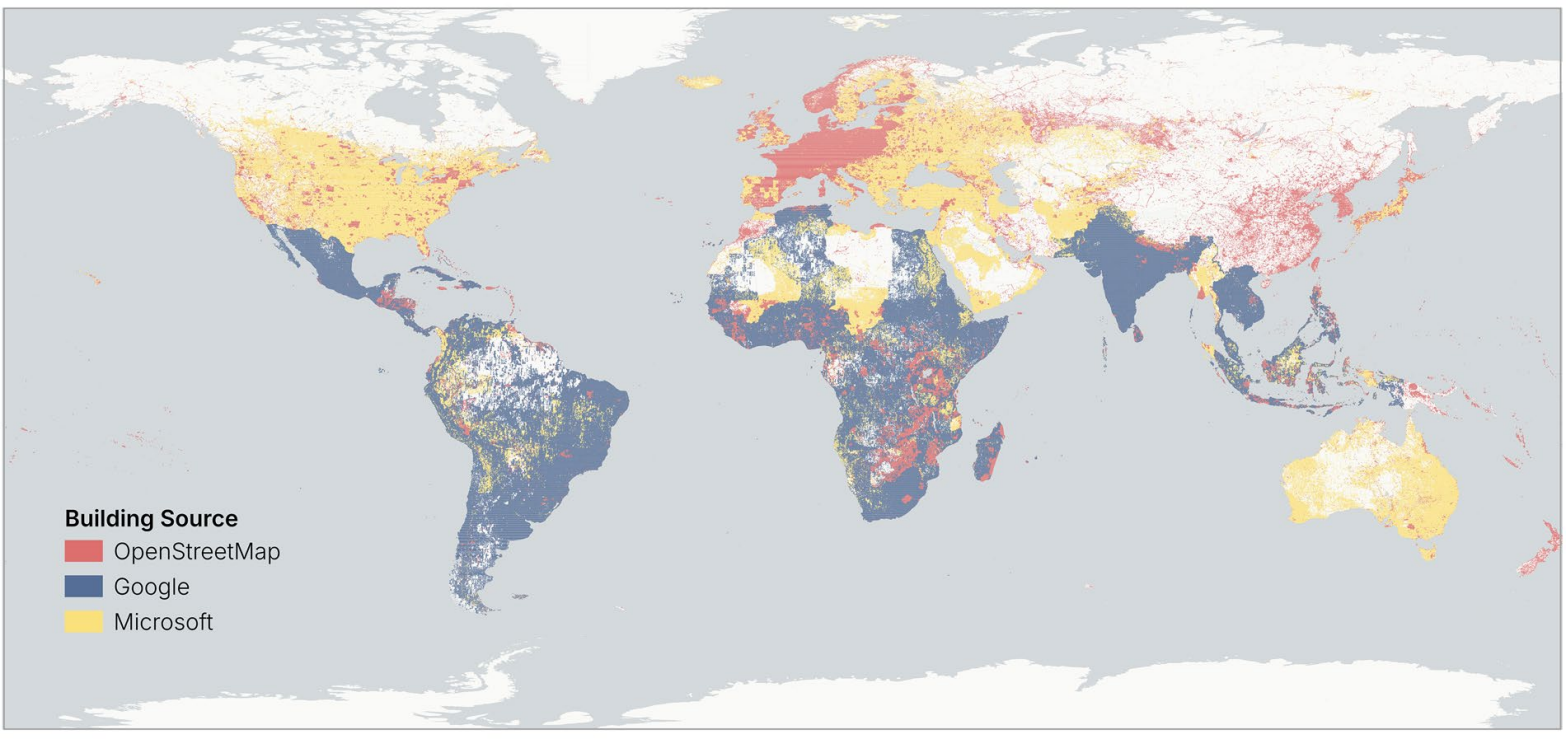
Prevalence of the building sources in the *OpenBuildingMap* dataset. The map is colored by the predominant building data source. ©Basemap by CARTO and OpenStreetMap contributors.

*OpenBuildingMap* is published on the GFZ Data Services^41^. In total, the resulting building database contains almost 2.7 billion buildings after filtering and conflation. In Table 1 the number of buildings per dataset and the share of height and occupancy attributes can be found. Figure 6 shows the main source of the dataset on a spatial scale. We can see that 23% of the buildings come from the *OpenStreetMap* dataset, which is most prevalent in urban areas, Western Europe and parts of Africa. This is in line with previous research^17,42^ and is a result of the mapping interests of individual mappers as well as the humanitarian endeavors. It is also the most prevalent in China, South Korea, North Korea and Taiwan, because the other two datasets do not exist there. 59% of the buildings are sourced from the *Google* dataset. As precendence is taken over the *Microsoft* dataset, we can see that it is prevalent in most of the global South. In the remaining areas (rural Northern America, Eastern Europe, the Middle East, Central Asia and Australia), the *Microsoft* dataset is most prevalent. The *Microsoft* dataset shares about 18% of the entire building dataset.

**Table 1:** Number of buildings in each dataset and the share of the height and occupancy attributes compared to the total number of buildings.

Dataset	Number of buildings	Height attribute	Occupancy attribute
OpenStreetMap	613M	510M (83.22%)	377M (61.43)%)
Google	1’598M	1’172M (73.35%)	458M (28.65%)
Microsoft	483M	344M (71.21%)	217M (44.96%)
Total	2’693M	2 026M (75.21%)	1’051M (39.03%)

### Building attributes

**Fig. 7:**
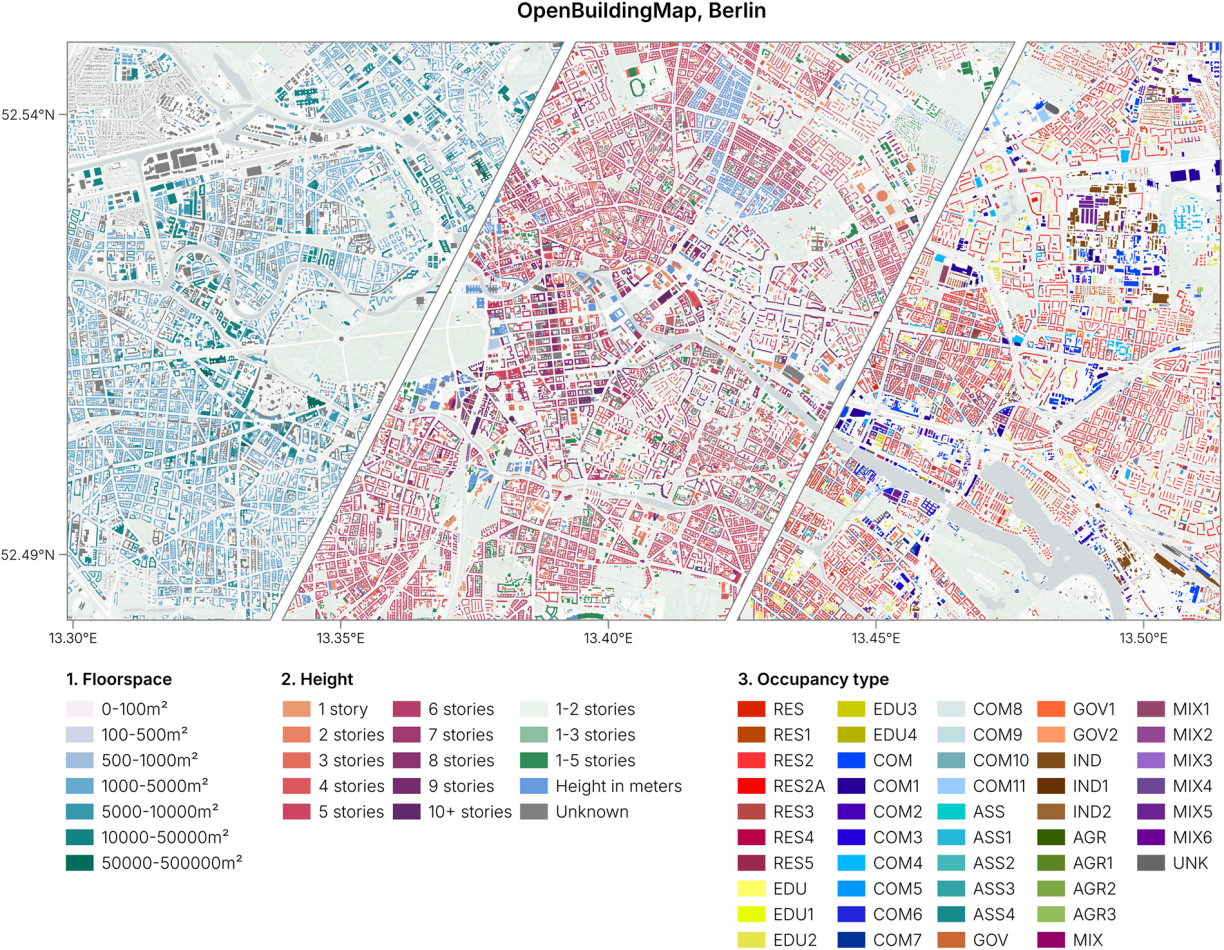
The building footprints in Berlin colored by floorspace, height and occupancy type. The full description of building occupancy codes is found in the data repository.

The dataset has four attributes: building height and/or number of stories (height), building occupancy type (occupancy), building floorspace (floorspace), and Quadtree ID (quadkey). A map showing the building occupancy type, height and floorspace is shown in Fig. 7. All attributes are explained in detail in this section. The height and occupancy attributes are described using the building taxonomy of the Global Earthquake Model^29^. This is an ontology that can be used for describing buildings, such as the material, year of construction and structural properties. It is a hierarchical structure, divided into segments that each delineate a topic. The topic can be further subdivided into multiple tags. A tag key is separated from a tag value by a colon (:) and tags of the same attribute are separated by a plus-sign (+). This system leaves some freedom in how to describe attributes in multiple ways. For example, the height attribute can be described as an exact number of stories, an interval of stories, or height above meters, or all of the three together.

#### Building height

The building taxonomy defines building height and number of stories as one attribute. The main segment is the number of stories above ground, which can be described with the H, HAPP or HBET tags. If the height is known, the H tag can be used to identify the number of stories (e.g. H:1 for a building with one story). If it is only known approximately, the HAPP attribute can be used. As the building taxonomy is often used in building stock models, where buildings are not described individually, but rather as a set of a certain type within a geographical area, it can be useful to describe a range of building stories. For that purpose, the HBET tag is used (e.g. HBET:2-3 for buildings between two and three stories). Alternatively, the height above ground in meters can be identified with the HHT tag (e.g. HHT:10.0 for a building that is ten meters tall). The number of stories below ground can be specified with HBEX, HBAPP and HBBET, respectively signifying the exact number of stories below ground, an approximation and a range. If there are multiple values, they can be combined with a plus-sign in between them (e.g. H:1+HBEX:1 for a building with one story and a basement). Exact details of the height tags can be found in the building taxonomy report^29^.

Table 2 shows the mapping of building height values of the different datasets to the building taxonomy. The *OpenStreetMap* dataset has an elaborate convention to deal with the number of stories and the height of a building as well. The building:levels and roof:levels tags summed are translated into the building taxonomy H tag. A dedicated tag is used to define the lowest floor level, if it is elevated above the ground. The building:min_level tag is used to describe the number of stories between the ground and the first actual building level. If this tag exists for a building, it has to be subtracted from the total value (e.g. a building with building:level=3 and building:min_level=1 will result in a building taxonomy H:2 tag). The building:levels:underground tag is used to describe the number of stories underground, similar to the HBEX building taxonomy tag. The building height in *OpenStreetMap* is described using the height tag. Again, if a building is elevated above ground, it is defined by the min_height tag, that is subtracted from the total height to calculate the HHT value.

**Table 2:** Translation of the height from the input datasets into the building taxonomy height attribute.

Input	Resulting height attribute
OpenStreetMap: building:levels = 2, roof:levels = 1	H:3
OpenStreetMap: building:levels = 5, building:min_levels = 1	H:4
OpenStreetMap: building:levels = 4, height = 16, building:levels:underground = 2	H:4+HBEX:2+HHT:16.00
Microsoft: height = 5.0	HHT:5.00
*GHSL*: height class = 6-15m	HBET:1-5

The *Microsoft* dataset has a height attribute, that can used unaltered for the HHT tag. The attribute only exists for parts of the *Microsoft* dataset, mainly in Europe, North America and Australia. The *Google* dataset does not include any height information.

For buildings without height information, an estimation is derived from *GHSL*, that separates multiple building classes (<3 m, 3-6 m, 6-15 m, 15-30 m, >30 m). As we have seen in Fig. 4, we can estimate with 95% certainty that buildings with the lowest value (<3 m) in *GHSL* translates to one or two building stories, and therefore are tagged with HBET:1–2. Buildings with the value 3–6m are tagged with HBET:1–3 and buildings with the value 6–15 are tagged with HBET:1–5. Buildings with a higher value in *GHSL* are not tagged with any height tag, because the inter-story height varies too much to provide an estimation of the number of stories for buildings higher than 15 meters.

**Fig. 8:**
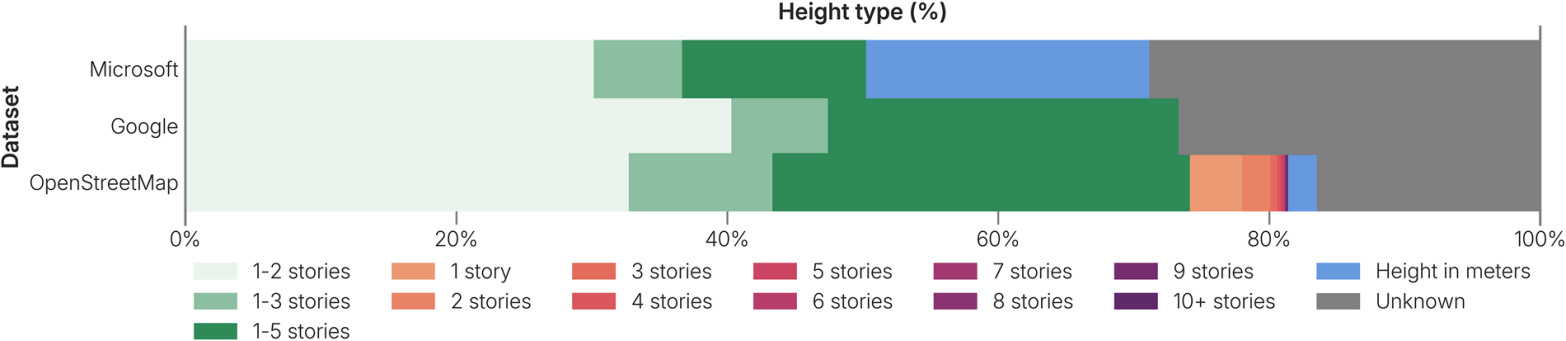
The share of height attributes in each of the datasets. In green shades, the share of buildings with a number of stories range is shown, in blue the height in meters, in red shades the exact number of stories and in gray the unknowns.

We can see in Table 1 that more than 75% of the building dataset include a height attribute. The source of the height can be found in Fig. 8. Most height attributes stem from *GHSL*. *OpenStreetMap* has the highest share of buildings with a height attribute, while the *Microsoft* dataset has the least. Approximately ten percent of the height attributes in the *OpenStreetMap* dataset originate from the *OpenStreetMap* building tags, while about thirty percent of the *Microsoft* dataset is derived from its own attribute.

#### Building occupancy type

The building taxonomy identifies eight main occupancy types: residential (RES), commercial and public (COM), mixed use (MIX), industrial (IND), agricultural (AGR), assembly (ASS), government (GOV) and education (EDU). If an occupancy is unknown, the UNK tag is used, and if it is any other occupancy type, OCO is used. Each of the main occupancy types could be divided into sub-types. For example, if it is known that a building is an apartment building, RES2 is used. If it is known that this apartment building has 50+ units, RES2F is used. The full description of the building taxonomy occupancy attribute can be found in the building taxonomy report^29^.

The occupancy attribute is solely based on the information found in *OpenStreetMap*. For each building footprint, all tags that are relevant to deduct the occupancy type are retrieved in and around the building using a spatial intersection. For *OpenStreetMap* buildings, the tags of the building itself are retrieved too. This creates a list of tags that provide information about the occupancy of the building. We are mapping *OpenStreetMap* tags to occupancy types of the building taxonomy. For example, the tag building=school (found as tag of a building) and amenity=school (found as tag of a land use) both resolve to the EDU occupancy type. The complete mapping is provided in the data repository. Certain occupancy types are expected to contain many different functions, such as public transport stations, airports or hospitals. In these cases, the defining function gets the priority and the other occupancy types are ignored. A list of such overriding occupancy types is found in the data repository.

**Fig. 9:**
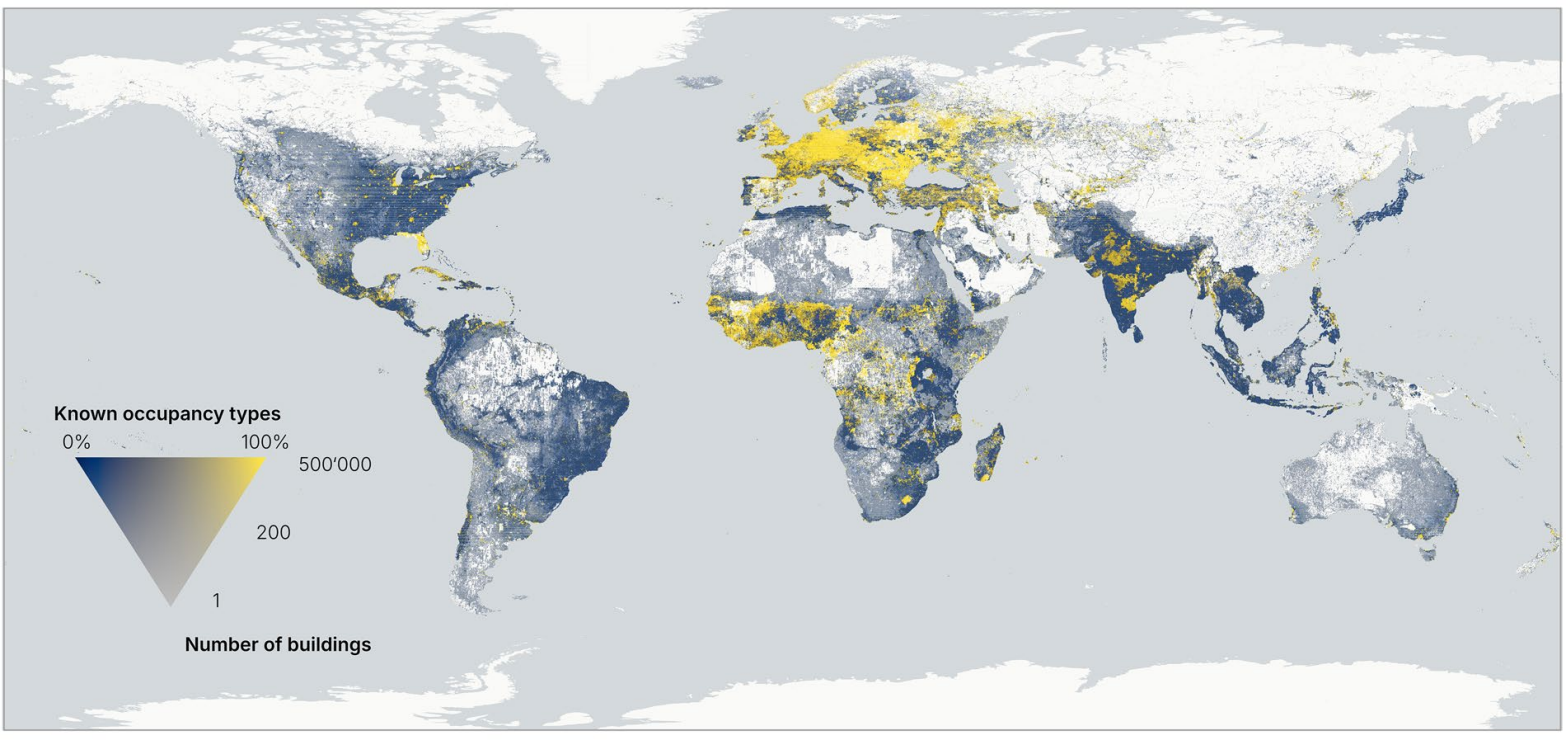
The percentage of buildings with known occupancy type over the total number of buildings. ©Basemap by CARTO and OpenStreetMap contributors.

In total, we are able to estimate the occupancy type for about 40% of the buildings (see Table 1). For *OpenStreetMap* buildings, this number is more than 60%, while for *Google* it is less than 30%. Figure 9 shows that the share of known occupancy types can differ strongly on a spatial scale. Some patterns can be observed: (1) the occupancy type can be better estimated in highly populated areas. We can observe that in the USA, where the patches of areas with a high share of known occupancy types aligns with densely populated areas. (2) Europe and West Africa have the highest share of occupancy types. (3) Some areas are well-covered, likely because of good land use coverage, that spans large areas. We can observe this in India, where the high share of known occupancy types does not necessarily follow whether the area is densely populated.

We can also compare the total number of buildings in the *OpenBuildingMap* dataset of each major occupancy type to the total number of buildings with equivalent tags in *OpenStreetMap* (see Table 3). The biggest increase can be found in the MIX occupancy type and the smallest increase in the COM occupancy type. The MIX and COM occupancy types are related to each other as most MIX buildings are a combination of residential and commercial buildings. There are between five and ten times more buildings for all other building occupancy types. The lion’s share of the buildings are tagged with a residential occupancy.

**Table 3:** Share of occupancy types in the *OpenStreetMap* and *OpenBuildingMap* datasets.

Occupancy	OpenStreetMap	OpenBuildingMap
Residential (RES)	93.0M	952.4M
Commercial and public (COM)	11.2M	14.8M
Mixed use (MIX)	33.8K	6.6M
Industrial (IND)	3.0M	23.2M
Agricultural (AGR)	4.1M	40.8M
Assembly (ASS)	612K	2.3M
Government (GOV)	356K	3.5M
Education (EDU)	1.5M	7.5M

The share of occupancy types in the *OpenStreetMap* dataset is estimated by summing building tags that are equivalent to the major occupancy tags in the *OpenBuildingMap* dataset. The building tags used used for the *OpenStreetMap* values can be found the data repository.

#### Usable floorspace

We estimated the usable floorspace as 70% of the building footprint area times the number of stories. The floorspace is only calculated if the number of stories is exactly known. The average height of one story depends not only on the region, but also on the building occupancy type and year of construction. As there is too much variation in inter-story height^36^, the floorspace attribute is not estimated for buildings with only a height in meter. If the number of stories is a range, as calculated from *GHSL*, the floorspace is not calculated either, as there is too much variation. For these reasons, only buildings from the *OpenStreetMap* dataset contain a floorspace attribute, in total 32 million buildings.

#### Quadkey

We computed the zoom-level 18 Quadkey at the centroid of each geometry. A Quadkey at this zoom-level is approximately 150 x 150 meters in size at the equator.

#### Additional files

There are five files that support the data publication. These can also be downloaded from the GFZ Data Services. The first file (A_Osmium_mapping.yaml) contains the Osmium mapping that was used to create the initial database with *OpenStreetMap* data. The second file (B_buildings_and_PoI_tags.csv) contains a mapping between *OpenStreetMap* tag values and the resulting GEM Taxonomy tag. In the third file (C_occupancy_types.csv), the explanation of each of the occupancy types is found. The fourth file (D_overriding_occupancies.csv) contains the list of overriding occupancy types. The last file (E_KullbackLeibler_cities.csv) contains the completeness index and Kullback-Leibler divergence of the three biggest cities in each country, that have at least 100.000 inhabitants.

## Technical Validation

### Completeness compared to the *GHSL* dataset

**Fig. 10:**
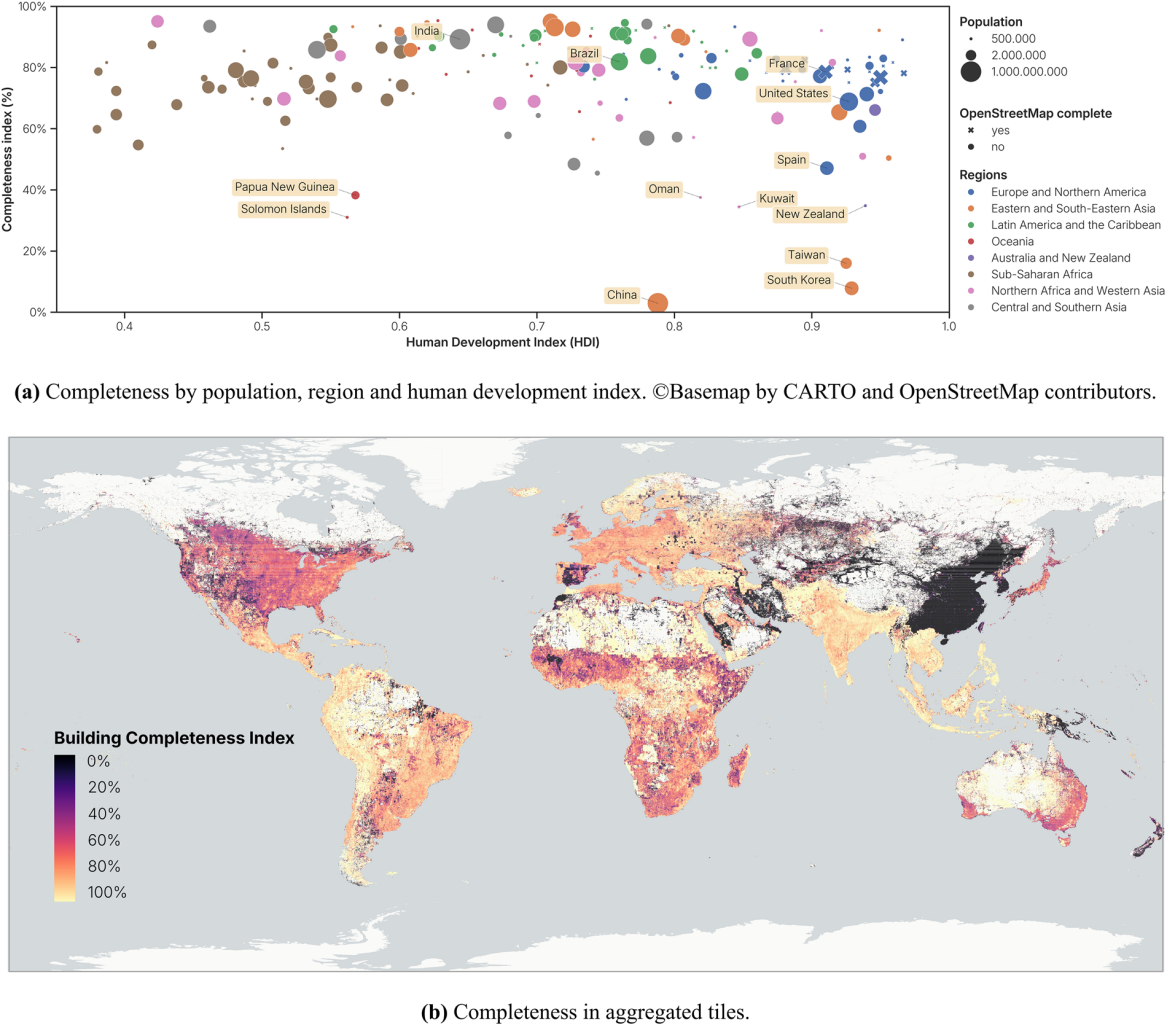
Completeness of the *OpenBuildingMap* dataset: number of tiles with buildings in the *OpenBuildingMap* dataset compared to number of tiles in the *GHSL* dataset.

The completeness of *OpenBuildingMap* has been computed using two indexes. A country completeness index has been calculated on country-level, where the total share of tiles with buildings was calculated over the tiles with built area according to *GHSL* (Fig. 10a). We know that the *GHSL* layer overestimates the built area, therefore we need reference countries that we can assume are complete in the *OpenBuildingMap* dataset. The countries marked with an X in the figure are complete in *OpenStreetMap* (therefore also in *OpenBuildingMap*) and are used as reference. The reference countries have a completeness index of around 70% or more. Most countries have a very similar completeness index as the reference countries, so we can assume that they are (nearing) completeness too. A tile completeness index has been computed on a zoom-level 12 Quadtree grid, to observe spatial patterns inside countries (Fig. 10b).

#### Australia and New Zealand

Australia has a high completeness index towards the interior. In the more populated coastal areas the completeness index is lower. New Zealand on the other hand, is largely incomplete.

#### Central and Southern Asia

India, Bangladesh, Pakistan and Afghanistan are largely complete. Iran is only complete in the more populated northern areas. Other areas are only sparsely mapped.

#### Eastern and South-Eastern Asia

The biggest blind spots in the dataset are found in this region. The dataset is near-empty in China, South Korea and Taiwan. Not only is little mapped in *OpenStreetMap* in the countries, but the *Google* and *Microsoft* datasets cover these regions either. This is opposed to other countries in South-East Asia, that have a very high completeness index and are likely mostly complete.

#### Europe and Northern America

Europe is largely complete, with the exception of Spain. Also, few patches remain in other countries spread over the region, where there is no *OpenStreetMap* data and the *Microsoft* data does not exist, likely because of lack of good satellite data. Canada and the United States are best mapped in the urban areas, where *OpenStreetMap* is most prevalent. Otherwise, the dataset is likely not fully complete, but not missing many buildings overall.

#### Latin America and the Caribbean

The region has overall a very high coverage in each country. The western side of South America has a high completeness index, as well as Central America and the Caribbean. The eastern side of South America is likely mostly complete, but lacking some information in the north.

#### Northern Africa and Western Asia

The completeness varies greatly within countries. For example, the northern (populated) area of Morocco is largely complete, while the southern area is not. the same holds for Saudi Arabia. The least complete are Oman and Kuwait.

#### Oceania

Most islands in Oceania have a very high completeness index, as they are well mapped in *OpenStreetMap*. Papua New Guinea and the Salomon Islands are exceptions and are incomplete.

#### Sub-Saharan Africa

Middle Africa has a high completeness index. Other areas, like North Africa, have a lower index, but are likely approaching completeness. One patch in Mali is very incomplete. This is where the *Google* dataset has no data and the *Microsoft* dataset is most prevalent (see Fig. 6).

In Fig. 10a no clear pattern can be found in the building completeness of *OpenBuildingMap*, when grouped into the regions (colours) or compared to the Human Development Index (y-axis). This is not the case for the *OpenStreetMap* dataset, which has the best coverage in the regions with the highest HDI^17^. While a clear pattern within countries isn’t visible in the higher-resolution completeness map in Fig. 10b, some pattern can still be observed. In some countries (USA, Japan, Morocco), the coverage is higher in urban regions and is lower in rural regions. In other regions, the exact opposite can be seen (Australia, Sahara dessert). In most countries, such pattern is not visible at all.

### Distribution of the occupancy type

**Fig. 11:**
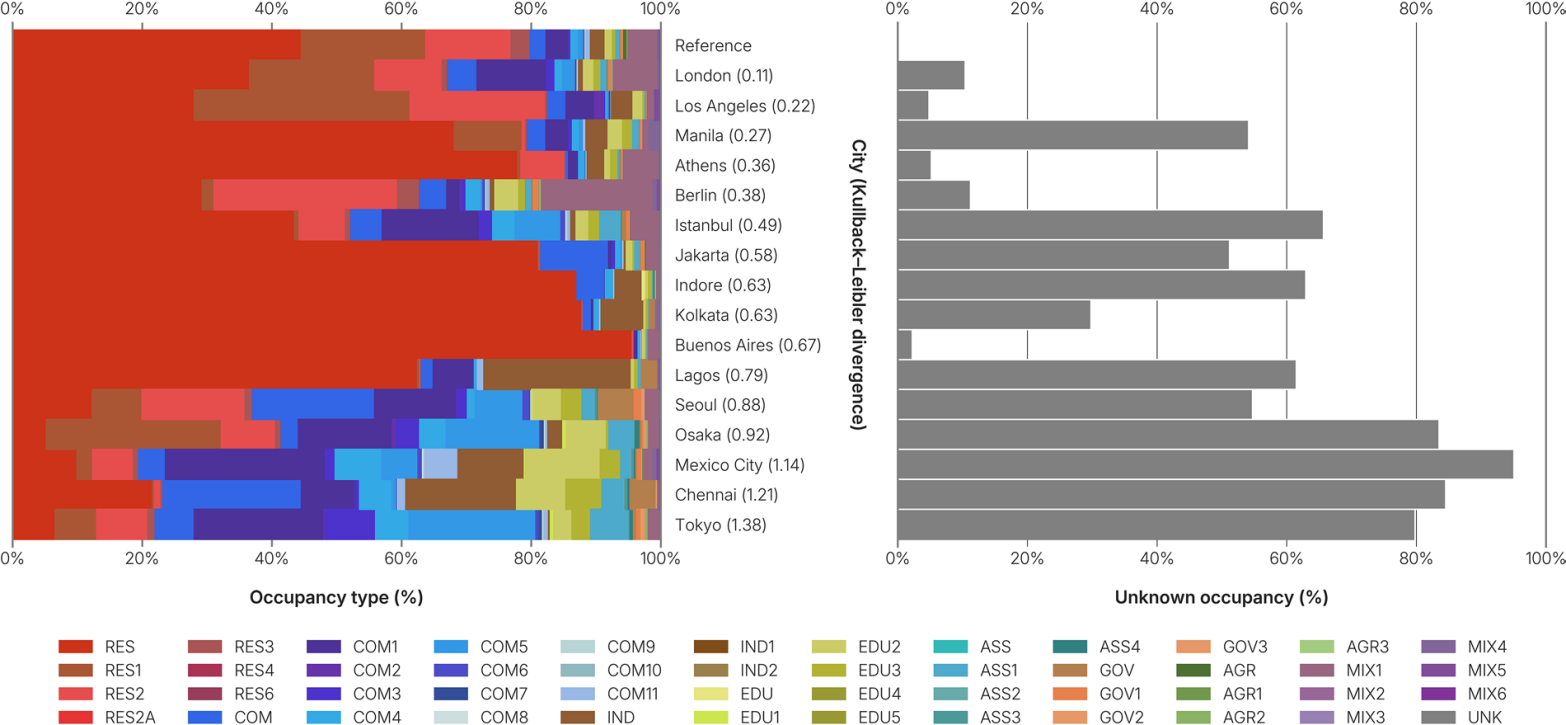
The left frame shows the distribution of occupancy types in 16 cities. The description of the building occupancy codes can be found in the data repository. The cities are ordered by the similarity to a reference distribution, or the Kullback-Leibler divergence (value in parentheses). The reference distribution is based on six cities (Amsterdam; Chicago; Vienna; Yaoundé; Singapore; Paramaribo). The right frame shows the percentage of unknown occupancy types in the 16 cities.

As one can not simply validate the individual occupancy types of buildings without reference data, instead the distribution of the data within some region has been compared with a reference distribution. For that, the Kullback-Leibler (KL) divergence has been used. For six reference cities (Amsterdam, Chicago, Vienna, Yaoundé, Singapore, Paramaribo), the distribution of occupancy types has been calculated and normalized. An average distribution of all six cities was computed after the normalization, to make sure each city was equally important for the final distribution. This was used as the input reference distribution for the Kullback-Leibler divergence. In turn, the divergence has been calculated for sixteen cities, spread around the world, ordered by low to high divergence: London (0.11), Los Angeles (0.22), Manila (0.27), Athens (0.36), Berlin (0.38), Istanbul (0.49), Jakarta (0.49), Indore (0.63), Kolkata (0.63), Buenos Aires (0.67), Lagos (0.79), Lagos (0.79), Seoul (0.88), Osaka (0.92), Mexico City (1.14), (Chennai (1.21), Tokyo (1.38). (see Fig. 11). A value closer to zero could indicate a more likely real distribution of occupancy types. Even though the share of buildings for which the occupancy is unknown is not included in the KL divergence, the four cities with the highest share of unknowns are also having the highest divergence. Buenos Aires has only very little unknown values, however it is likely that the reference distribution does not accurately represent reality, as can be found through the low KL divergence value and the high number of residential units compared to any other occupancy type. The Kullback-Leibler divergence is not perfect, as we can see that Berlin, that has a high share of mixed buildings, should be one of the cities with the best data available, but is instead having a lower value than Athens, where the quality of the *OpenStreetMap* information is lower. Nevertheless, the divergence captures a general trend of occupancy quality, even more so in combination with the percentage of unknown occupancy in the city.

### Validation using the Greek and Slovene cadaster

**Fig. 12:**
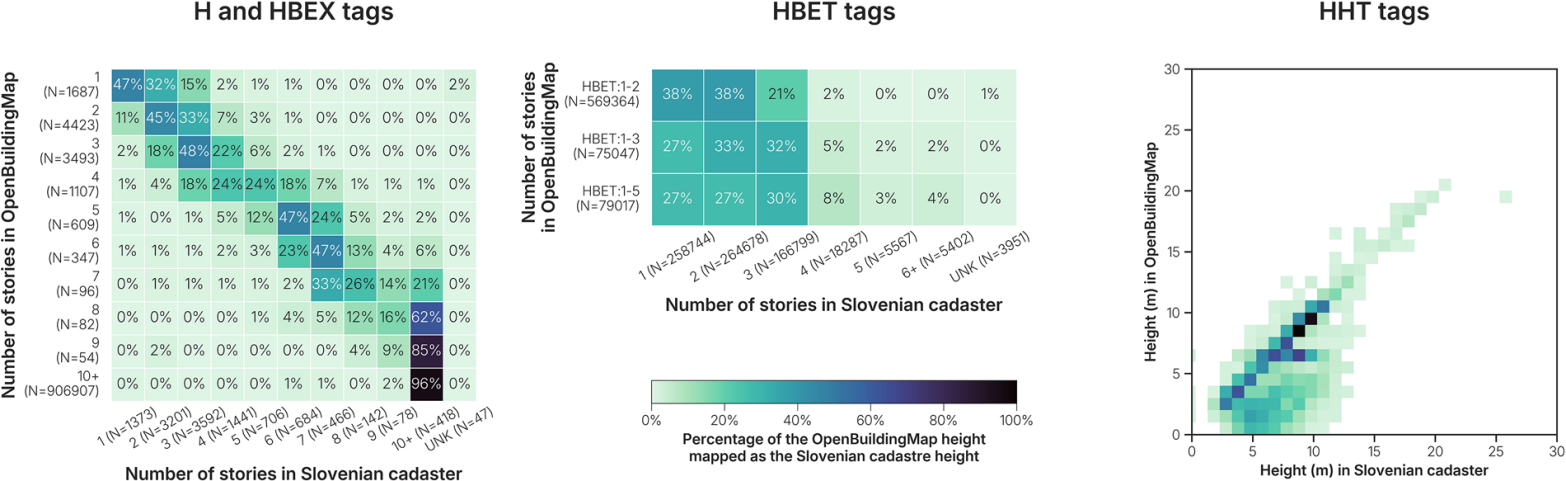
Comparison between the height information in the Slovenian cadaster and the *OpenBuildingMap* height tags. The number of stories in the Slovenian cadaster also includes the basement level, if it exists. As this is usually not reported in *OpenStreetMap* and the number of stories sourced from the settlement layer is related to building height, there could be a mismatch of maximum one story. The source of the H and HBEX tags is always *OpenStreetMap*. The source of HBET tags is always the *GHSL* dataset and the source of the HHT tag is either *OpenStreetMap* or *Microsoft*.

We have compared the height and number of stories found in the cadaster with the *OpenBuildingMap* dataset (see Fig. 12) in Slovenia. The cadaster building height is retrieved by subtracting the building characteristic height from the maximum height of the building. The number of stories is a value including basements. We have separated the three methods to estimate height or number of stories (number of stories as single value, number of stories as range and height as single value) and computed the accuracy of each. Additionally, the Root Mean Square Error (RMSE) and Mean Absolute Error (MAE) have been computed for each method. In the case of the building range, the value within the range that is closest to the value in the cadaster has been taken as the estimated value. If we consider a building with the HBET:1-3 tag and the building has 2 stories according to the cadaster, the ground truth lies within the estimated range, so the error is 0 stories. If the cadaster reports 4 stories instead, we use the upper bound of the range (3 stories) as the estimated value, resulting in an error of 1 story. The comparison can have minor discrepancies: it is not possible with satellite imagery to discover if a basement is present. It is also difficult to map the number of basements in *OpenStreetMap*, as they are often not visible from the outside. Lastly, the buildings may consist of multiple overlapping building parts, which could result in discrepancies too. 43% of the number of stories as single value (H/HBEX tags) are exactly the same as in the cadaster. 83% of the buildings with these tags are at most 1 building story off (RMSE=1.28; MAE=0.83). 81% of buildings with a building range (HBET tag) are correctly identified and 98% have at maximum one building story more in the cadaster than the upper end of the range (RMSE=0.54, MAE=0.22). 48% of the buildings with a height in meter (HHT tag) have at most 1 meter of difference with the building cadaster (RMSE=4.12, MAE=2.31). 91% of the buildings have at most 3 meters of difference. Given the limitations with detecting basements, the height attribute of OpenBuildingMap is very similar to the Slovenian dataset.

Greece is an interesting case, as the majority of the building footprints in *OpenBuildingMap* do not come from the *OpenStreetMap* dataset, but from the *Microsoft* dataset. The data quality is therefore expected to be slightly worse than in case of a complete coverage of *OpenStreetMap*, as there is simply less information per building available. The cadaster dataset that is used as reference is not comprehensive, but includes large parts of the country.

**Fig. 13:**
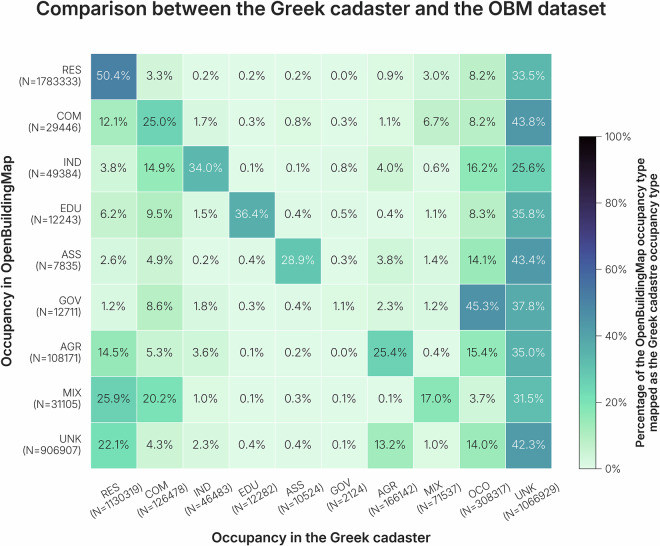
Comparison of the Greek cadaster with the *OpenBuildingMap* occupancy types. The values of the cadaster have been mapped to align with the building taxonomy occupancy types.

The results of the validation using the Greek cadaster can be found in Fig. 13. The validation proved to be difficult to assess. (1) The cadaster holds a large amount of unknown values. Between 25% and 45% of each of the occupancy types found in *OpenBuildingMap* have no equivalent in the cadaster. (2) The cadaster holds a large amount of unidentified occupancy types, that may be outdated or uninformative (such as uncovered area, other space and special use). For example, military bases are classified as a type of governmental building in the *OpenBuildingMap* dataset. However, most governmental buildings (including military bases) are not tagged as governmental in the Greek cadaster, but rather are tagged as other space in the Greek cadaster. Therefore, even though in the figure it seems that most governmental buildings are wrongly classified in the *OpenBuildingMap* dataset, but in reality they are not classfied at all in the cadaster.

One of the most misclassified building type are residential buildings tagged as agricultural in *OpenBuildingMap*. These are buildings that are situated in agricultural areas. In such cases, building footprint coverage in *OpenStreetMap* is minimal, while the land use has already been identified at a large scale on the platform (agricultural). The buildings do exist in the *Microsoft* dataset, so the only information we have about the buildings is that the land use is agricultural and they are therefore tagged as such. Another false classification happens with the mixed building types. 30% of the buildings tagged as mixed in *OpenBuildingMap* are tagged as residential in the Greek cadaster. The official definition of the residential building class in the Greek cadaster is >80% residential, meaning it could be fully residential, but also a mixed building. The commercial buildings tagged as mixed are mostly wrongly tagged as mixed, but exist in a mixed-use neighborhood, for example bank buildings. A cadaster could be seen as the authoritative dataset for building information. Upon closer inspection, we can see that the *OpenBuildingMap* dataset shows a similar accuracy to the cadaster and that many misclassifications come from a difference in definition, rather than errors in the classification. In addition, *OpenBuildingMap* has more building geometries in the area, as well as more buildings classified with an occupancy. So, even in countries with an existing cadaster, the *OpenBuildingMap* dataset proves to be of value.

Information about building occupancy types primarily comes from the *OpenStreetMap* dataset, either through building attributes, land use or points of interest. The growing volume of the *OpenStreetMap* dataset will improve the accuracy of *OpenBuildingMap* occupancy type classification. Likewise, new and more detailed datasets derived from Earth observation can be easily integrated in *OpenBuildingMap* and further enhance the dataset. The occupancy type can also be estimated by integrating data from urban morphology or building geometry, processed using artificial intelligence algorithms. Previous research has classified buildings into residential and non-residential buildings using deep learning^43^, but one could go a step further and predict building occupancy according to the eight main occupancy classes, or the 50+ sub-classes of the building taxonomy. Machine learning can be trained on field-validated data or reference datasets such as the buildings with occupancy types that are known in the *OpenBuildingMap* dataset. Similarly, building height estimation could be refined through the use of extra building height source datasets, machine learning, or the use of Gaussian mixture models. Urban patterns, taking into account contextual factors such as the urban-rural distinction, can strongly influence building typologies and heights. For example, we can expect a general trend that buildings in a rural setting are lower on average than buildings in city centers.

## Usage Notes

The *OpenBuildingMap* dataset is published as separate GeoPackage files on a zoom-level 6 Quadkey grid. On the website https://www.openbuildingmap.org/download, the tile grid is displayed. A user can click on a tile to download the file.

### Limitations and uncertainty

The *OpenBuildingMap* dataset varies regionally in quality and completeness of attributes, as height and occupancy. Ground-truth data is lacking, so it is not possible to quantify such measures. However, it is possible to provide some information that can give a basic idea on the quality and completeness. Therefore, each tile has been supplemented with the completeness index, as well as the completeness of the occupancy, height and floorspace attributes. Also the number of buildings and the size of the GeoPackage is shown. On top of that, the Kullback-Leibler divergence and percentage of unknown occupancy types are calculated for the three biggest cities in each country with at least 100.000 inhabitants. In total, the value is calculated for 447 cities. The values can serve as an indication of the quality of the occupancy information of the dataset, and can be found the data repository.

The building footprint geometries have not been subjected to quality testing in this research. The geometries were not modified after they were taken from the source datasets, therefore, the quality of the footprints can be inferenced from the quality of the footprints in each of the source datasets. The precedence of the building conflation was based on the quality of the building footprints, therefore we are confident that locally the dataset contains the best available footprint geometry. The number of buildings sourced from each of the datasets is stated in the tile download too, so this can be used as a quality indicator (i.e. more *OpenStreetMap* buildings can be an indicator for better quality data). Despite the use of a relatively simple conflation procedure, it proves to be efficient and adequate. In the future, more advanced approaches, including AI-based methods, could be explored, provided they offer a clear scientific benefit in terms of data quality while maintaining a reasonable carbon footprint.

Some error types we are not able to detect, such as incorrectly placed buildings in *OpenStreetMap* or the use of old satellite imagery in the *Google* or *Microsoft* datasets. It is also difficult to discover incorrect or outdated attribute information. Such details require extra research, for example by using the *Ohsome* dashboard^17^, that can identify if a region is up-to-date in *OpenStreetMap*. The input sources update frequently (*OpenStreetMap* updates continuously and the other two datasets have multiple new releases per year) and building the *OpenBuildingMap* dataset is mostly automatic. Therefore, the dataset can be rebuilt again any time with the updated data.

## Data Availability

*OpenBuildingMap* is published on the GFZ Data Services (10.5880/GFZ.LKUT.2025.002). The data is distributed as GeoPackage files on a zoom-level 6 Quadtree grid. The Quadkey is used to name the file. All files have one layer named building, containing the building data. On https://www.openbuildingmap.org/download the zoom-level 6 tiles are displayed on an interactive map, with links to the respective files of the tiles and basic statistics such as the percentage of occupancy types known.

## Code availability

The import of *OpenStreetMap* data to *PostGIS* database has been done using Imposm and a custom mapping file, published to our database-osmreplication repository. The import of *Google* and *Microsoft* data to a *PostGIS* database has been done with the all-in Python package. The building conflation was achieved with the obm-conflation Python package and the addition of attributes was done with the building-initializer, using the rules defined in rabotnik-rules. All analysis and figures have been published in the obm-conflation analysis directory.
